# F-Prostaglandin receptor regulates endothelial cell function via fibroblast growth factor-2

**DOI:** 10.1186/1471-2121-11-8

**Published:** 2010-01-21

**Authors:** Margaret C Keightley, Pamela Brown, Henry N Jabbour, Kurt J Sales

**Affiliations:** 1 MRC Human Reproductive Sciences Unit, The Queen's Medical Research Institute, 47 Little France Crescent, Edinburgh, EH16 4TJ, UK

## Abstract

**Background:**

Prostaglandin (PG) F_2α _is a key regulator of endometrial function and exerts its biological action after coupling with its heptahelical G protein-coupled receptor (FP receptor). In endometrial adenocarcinoma the FP receptor expression is elevated. We have shown previously that PGF_2α_-FP receptor signalling in endometrial adenocarcinoma cells can upregulate several angiogenic factors including fibroblast growth factor-2 (FGF2). In the present study, we investigated the paracrine effect of conditioned medium produced via PGF_2α_-FP receptor signalling in endometrial adenocarcinoma cells stably expressing the FP receptor (Ishikawa FPS cells), on endothelial cell function.

**Results:**

Conditioned medium (CM) was collected from FPS cells after 24 hrs treatment with either vehicle (V CM) or 100 nM PGF_2α _(P CM). Treatment of human umbilical vein endothelial cells (HUVECs) with P CM significantly enhanced endothelial cell differentiation (network formation) and proliferation. Using chemical inhibitors of intracellular signalling, we found that P CM-stimulated endothelial cell network formation was mediated by secretion of endothelial PGF_2α _and activation of endothelial FP receptors, following FGF2-FGFR1 signalling, phosphorylation of ERK1/2 and induction of COX-2. Whereas, P CM stimulation of endothelial cell proliferation occurred independently of PGF_2α _secretion via an FGF2-FGFR1-ERK1/2 dependent mechanism involving activation of the mTOR pathway.

**Conclusions:**

Taken together, we have shown a novel mechanism whereby epithelial prostaglandin F_2α_-FP signalling regulates endothelial cell network formation and proliferation. In addition we provide novel in vitro evidence to suggest that prostaglandin F_2α _can directly regulate endothelial cell network formation but not endothelial cell proliferation. These findings have relevance for pathologies where the FP receptor is aberrantly expressed, such as endometrial adenocarcinoma, and provide in vitro evidence to suggest that targeting the FP receptor could provide an anti-angiogenic approach to reducing tumour vasculature and growth.

## Background

Endometrial adenocarcinoma, originating from the glandular epithelial cells of the uterine endometrial lining, is one of the most prevalent cancers amongst women in the Western world [1,2]. It is a disease which particularly occurs in post menopausal women and recent evidence suggests that mutations in oncogene expression may play a role in the etiology of the disease [3]. Data generated in our laboratory and others have ascertained a role for the cyclooxygenase (COX)-prostaglandin (PG) axis in the regulation of endometrial adenocarcinomas by increasing cell proliferation and the secretion of angiogenic growth factors [4,5]. This is similar to other cancers where over-expression of COX enzymes and biosynthesis of prostaglandins has been shown to promote cellular proliferation [6], inhibit apoptosis [7] and enhance angiogenesis [8]. However, the molecular mechanisms mediating the role of prostaglandins in regulating vascular function and angiogenesis are still poorly defined.

Angiogenesis is the process of endothelial cell sprouting from an existing vasculature towards cancer cells [9] and is required by any tumour larger than 2 mm in diameter [10]. The proposed mechanism of angiogenesis suggests that tumour cells secrete stimulatory factors which act in a paracrine manner on surrounding blood vessels, immune cells and fibroblasts to promote the proliferation, differentiation and migration of endothelial cells towards the stimulus [10,11]. These tumour stimulatory factors include vascular endothelial growth factor (VEGF-A) and fibroblast growth factor 2 (FGF2). In human endometrial adenocarcinomas VEGF-A and FGF2 expression and secretion are elevated [12-14] and both VEGF-A and FGF2 can stimulate angiogenesis in xenografts in vivo [15,16].

In a previous study we demonstrated elevated expression of the FP receptor, FGF2 and the FGF2 receptor 1 (FGFR1) in neoplastic endometrial epithelial and vascular cells and ascertained a role for the FGF2, produced by PGF_2α_-FP receptor signalling, on epithelial cell proliferation [12]. In this study we have shown that conditioned medium from PGF_2α _treated Ishikawa cells stably expressing the FP receptor (Ishikawa FPS cells), can increase endothelial cell differentiation (network formation) and proliferation. Treatment of Ishikawa FPS cells with PGF_2α _increases FGF2 secretion which in turn activates FGFR1 signalling in endothelial cells and induces the phosphorylation of extracellular signal-regulated kinase (ERK1/2), COX-2 expression and secretion of PGF_2α_. Following its release from endothelial cells, we show for the first time that, PGF_2α _promotes endothelial cell network formation in an autocrine/paracrine manner, via the endothelial FP receptor. By contrast, PGF_2α _is not involved in endothelial cell proliferation which we show to be regulated by FGF2-FGFR1 signalling via the mammalian target of rapamycin (mTOR) pathway. Taken together, our data highlight two molecular pathways by which PGF_2α_-FP receptor signalling can regulate endothelial cell function in endometrial adenocarcinomas.

## Results

### PGF_2α_-FP signalling mediates endothelial cell network formation and proliferation via FGF2-FGFR1 signalling

We previously demonstrated elevated expression of the FP receptor, FGF2 and FGFR1 in endometrial adenocarcinoma [12]. Using a neoplastic epithelial cell line stably expressing the FP receptor to the levels observed in endometrial adenocarcinoma (Ishikawa FPS cells), we ascertained a role for FGF2, produced by PGF_2α_-FP receptor signalling, on epithelial cell proliferation [12]. In addition, we found that FP receptor, FGF2 and FGFR1 co-localised within the vascular endothelial cells in endometrial adenocarcinomas suggesting that PGF_2α _may directly and indirectly regulate endothelial cell function [12]. To determine if the effects of PGF_2α_-FP receptor interaction in endometrial adenocarcinoma cells on endothelial cell function were mediated by FGF2, we used conditioned medium (CM) from Ishikawa FPS cells treated with vehicle or 100 nM PGF_2α _for 24 hours. The presence of FGF2 in CM from vehicle (V CM) and PGF_2α_-treated (P CM) Ishikawa FPS cells was confirmed by ELISA. Immunoneutralisation of P CM with FGF2 antibody significantly reduced FGF2 concentration in P CM [12].

**Figure 1:**
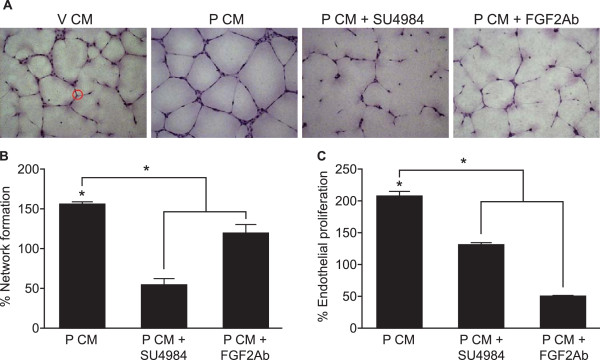
**The effect of PGF_2α _conditioned medium on endothelial network formation and proliferation**. (A) Representative image from one network formation assay in which HUVECs, plated on growth factor reduced matrigel, were incubated with V CM, P CM or P CM and SU4984 or FGF2 immunoneutralised CM (FGF2-Ab) for 16 hrs as described in the Methods. (B) Quantification of endothelial network formation and (C) proliferation using HUVECs stimulated with V CM, P CM or P CM in the presence of FGFR1 tyrosine kinase inhibitor (P CM + SU4984) or FGF2 immunoneutralised CM (P CM + FGF2Ab). Data are expressed as percentage increase over control V CM (where V CM = 100%, not shown) and presented as mean ± SEM. (* represents statistical significance P < 0.05).

To assess the effects of the CM on differentiation (network formation) and proliferation, assays were performed using HUVECs as a model system. Treatment of HUVECs with P CM significantly increased endothelial cell network formation (Fig. 1A and 1B, P < 0.05) and proliferation (Fig. 1C, P < 0.05) compared to V CM-treated cells. Treatment of HUVECs with P CM in the presence of the FGF2 receptor 1 (FGFR1) tyrosine kinase inhibitor (SU4984) or FGF2-immunoneutralised CM (FGF2-Ab), significantly reduced endothelial cell network formation (Fig. 1A and 1B; P < 0.05) and cellular proliferation (Fig. 1C; P < 0.05), confirming that these alterations in endothelial cell function were mediated by FGF2 in the P CM signalling through endothelial FGFR1.

**Figure 2:**
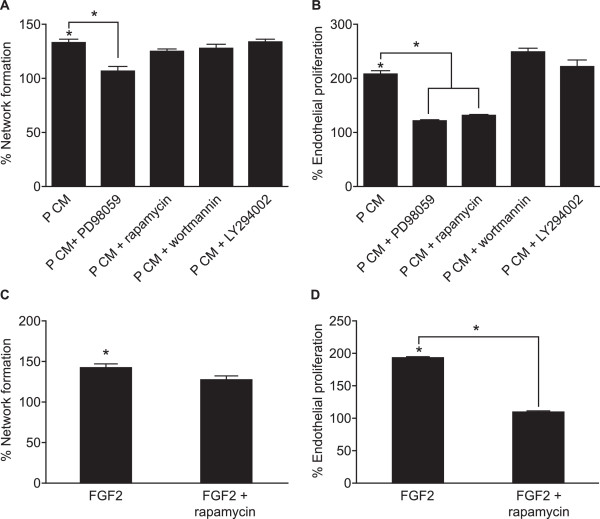
**Endothelial network formation and proliferation is mediated via the ERK1/2 pathway**. (A) Endothelial network formation and (B) proliferation in HUVECs treated with V CM or P CM in the absence/presence of inhibitors of ERK1/2 (PD98059), mTOR (rapamycin) or PI3K (wortmannin or LY294002). (C) Endothelial network formation and (D) proliferation in HUVECs treated with vehicle or recombinant FGF2 protein (50 ng/ml) in the absence/presence of rapamycin. Data are expressed as percentage increase over control V CM (where V CM = 100%, not shown) and presented as mean ± SEM. (* represents statistical significance P < 0.05).

Next we investigated the signal transduction pathways mediating the role of FGF2 in the P CM on network formation and proliferation. HUVECs were treated with P CM in the presence of cell signalling inhibitors of extracellular signal-regulated kinase (ERK1/2; PD98059), mammalian target of rapamycin (mTOR; rapamycin) or phosphoinositide-3-kinase (PI3K; wortmannin or LY294002). We found that the P CM-induced network formation was significantly inhibited by PD98059 but not rapamycin, wortmannin or LY294002 (Fig. 2A, P < 0.05). However, endothelial cell proliferation was inhibited by PD98059 and rapamycin but not wortmannin or LY294002 (Fig. 2B, P < 0.05).

We confirmed that endothelial cell proliferation but not network formation was mediated by FGF2-mTOR signalling using recombinant FGF2 protein. Treatment of HUVECs with recombinant FGF2 significantly increased network formation (Fig. 2C; P < 0.05) and proliferation (Fig. 2D; P < 0.05). Co-treatment of cells with recombinant FGF2 protein and rapamycin had no effect on network formation, compared to recombinant FGF2 peptide alone (Fig. 2C; P < 0.05). In contrast, rapamycin treatment significantly inhibited endothelial cell proliferation induced by the recombinant FGF2 protein (Fig. 2D; P < 0.05) confirming that endothelial cell proliferation was mediated by P CM via the FGF-FGFR1-mediated induction of the mTOR pathway.

### ERK1/2 phosphorylation is regulated by FGF2- FGFR1 signalling

**Figure 3:**
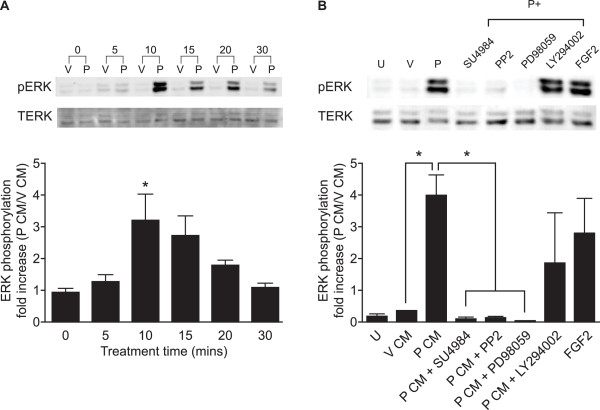
**The effect of conditioned medium on ERK1/2 signalling in HUVECs**. (A) HUVECs were treated with V CM (V) or P CM (P) for 0,5,10,15,20 and 30 minutes. (B) HUVECs were left untreated (U) or treated with V CM (V) or P CM (P) for 10 mins in the absence/presence of SU4984, PP2, PD98059 or LY294002. Cell lysates were subjected to immunoblot analysis using antibodies against phosphorylated ERK1/2 (top panel) and total ERK1/2 (bottom panel). A representative Western blot is displayed with a graph of semi-quantitative analysis of ERK1/2 phosphorylation determined as described in the Methods. (* represents statistical significance P < 0.05) Data are represented as mean ± SEM.

As ERK1/2 was involved in regulating both P CM induced endothelial cell network formation and proliferation, we investigated the effect of P CM on ERK1/2 phosphorylation. HUVECs were treated with V CM or P CM for 0, 5, 10, 15, 20 and 30 mins (Fig. 3A). Treatment of HUVECs with P CM significantly increased ERK1/2 phosphorylation in a time-dependent manner which was maximal after 10 mins of stimulation, compared to V CM (Fig. 3A; P < 0.05). Co-incubation of HUVECs with P CM in the presence of FGFR1 tyrosine kinase inhibitor (SU4984), c-Src inhibitor (PP2) or ERK1/2 inhibitor (PD98059) significantly reduced the P CM-stimulated phosphorylation of ERK1/2 to basal levels (Fig 3B, P < 0.05). However treatment of HUVECs with P CM in the presence of the PI3K inhibitor LY294002 did not significantly reduce the P CM phosphorylation of ERK1/2 (Fig. 3B). Similarly, co-incubation of HUVECs with P CM and the mTOR inhibitor, rapamycin, had no effect on ERK1/2 phosphorylation (data not shown). Treatment of HUVECs with recombinant FGF2 protein phosphorylated ERK1/2 to the levels observed with P CM (Fig. 3B).

### Conditioned medium from Ishikawa FPS cell treated with PGF_2α _induces endothelial COX-2

**Figure 4:**
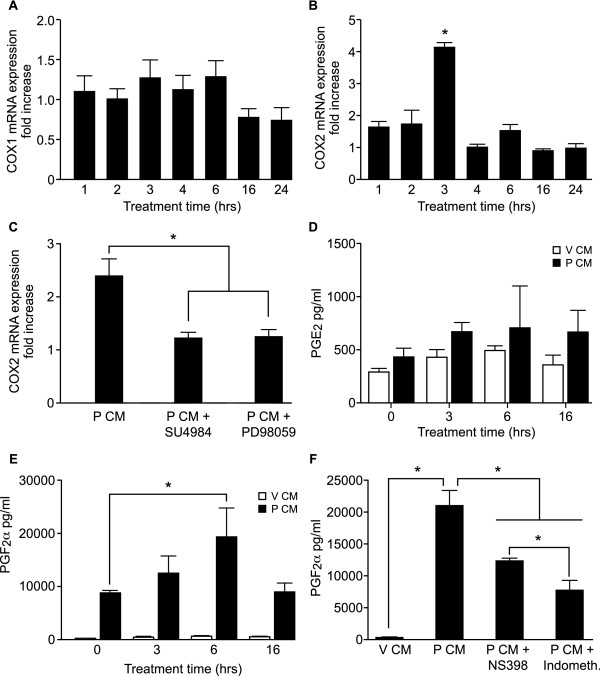
**Conditioned medium induces expression of COX-2 and prostaglandin F_2α _secretion**. Expression of (A) COX-1 and (B) COX-2 mRNA in HUVECs incubated with V CM or P CM for 1,2,3,4,6,16 and 24 hrs as determined by quantitative RT-PCR analysis. (C) COX-2 mRNA in HUVECs incubated with V CM or P CM for 3 hrs in the absence/presence of FGFR1 tyrosine kinase inhibitor SU4984 or ERK1/2 inhibitor PD98059 as determined by quantitative RT-PCR analysis. Data are expressed as fold increase over control V CM (where V CM = 1, not shown). HUVECs were incubated with V CM or P CM for 0, 3, 6 and 16 hrs and thereafter conditioned medium from HUVECs was collected and used for PGE_2 _(D) and PGF_2α _(E) ELISA analysis as described in the methods. (F) HUVECs were treated with V CM, P CM or P CM and NS398 or indomethacin (Indometh.) for 6 hrs after which the concentration of PGF_2α _in the HUVEC medium was determined by ELISA. Data are represented as mean ± SEM. (* represents statistical significance P < 0.05).

FGF2 has been shown to mediate angiogenesis via COX-2 in an in vivo model using rat sponge implants [17], hence we investigated the effect of P CM on the expression of COX-1 and COX-2 in endothelial cells. HUVECs were treated with V CM or P CM for 1, 2, 3, 4, 6, 16 and 24 hrs. We did not observe an alteration in the expression of COX-1 (Fig. 4A) at any of the time points investigated in response to P CM stimulation. However, we observed a significant increase in endothelial COX-2 expression at 3 hours following treatment with P CM (Fig. 4B; P < 0.05). This increase in COX-2 expression was inhibited by treatment with P CM in the presence of the FGFR1 inhibitor, SU4984 or ERK1/2 inhibitor, PD98059 (Fig. 4C; P < 0.05) indicating that COX-2 expression was regulated via the FGF-2-FGFR1-ERK1/2 signalling pathway.

Cyclooxygenase enzymes are responsible for the catalysis of arachidonic acid to prostaglandins (PG). We next investigated the secretion of endothelial PGE_2 _and PGF_2α _in response to CM treatment. HUVECs were treated with V CM or P CM for 0, 3, 6, and 16 hrs and the secretion of PGE_2 _(Fig. 4D) and PGF_2α _(Fig. 4E) was measured by ELISA. There was no significant difference in the levels of endothelial PGE_2 _secreted by HUVECs in response to CM at any time point tested (Fig. 4D). In contrast, the amount of endothelial PGF_2α _was elevated in HUVECs at 6 hrs following P CM treatment compared to both V CM treatment (6 hrs) and P CM at time 0 hrs (Fig. 4E; P < 0.05). To confirm the involvement of COX in the secretion of PGF_2α_, we treated HUVECs for 6 hrs with P CM in the presence of the specific COX-2 inhibitor NS398 or the general COX inhibitor indomethacin. We found that the P CM-induced secretion of PGF_2α _was significantly reduced by co-treatment of cells with NS398 (Fig. 4F; P < 0.05). Similarly, co-treatment of cells with indomethacin significantly reduced P CM-induced PGF_2α _secretion below the levels observed with P CM alone and P CM with NS398 (Fig. 4F; P < 0.05).

### The role of endothelial prostaglandin F_2α_-FP signalling in the regulation of endothelial cell network formation

**Figure 5:**
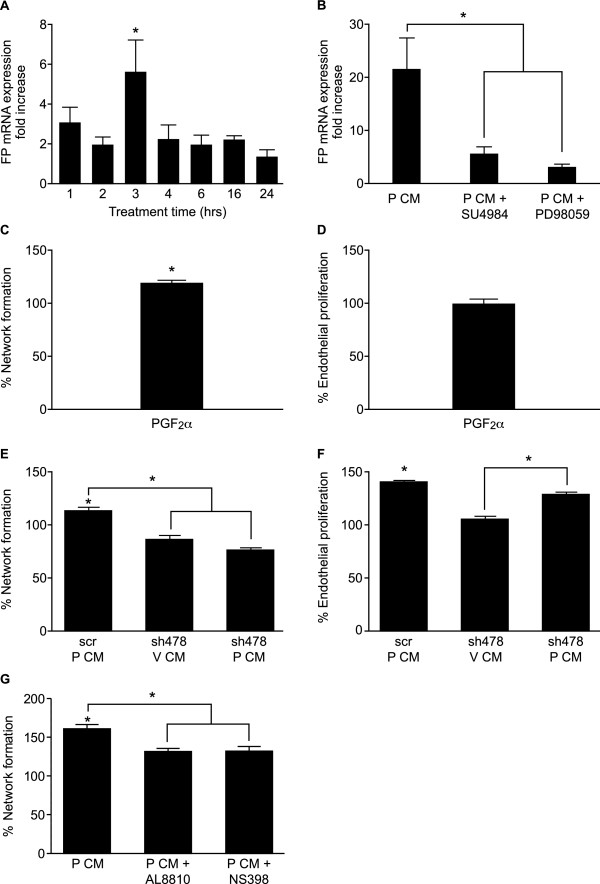
**Endothelial network formation is regulated by endothelial PGF_2α_-FP receptor interaction**. (A) Expression of FP receptor in HUVECs incubated with V CM or P CM for 1,2,3,4,6,16 and 24 hrs as determined by quantitative RT-PCR analysis. (B) FP receptor mRNA in HUVECs incubated with V CM or P CM for 3 hrs in the absence/presence of FGFR1 tyrosine kinase inhibitor SU4984 or ERK1/2 inhibitor PD98059 as determined by quantitative RT-PCR analysis. HUVECs were incubated with vehicle or exogenous PGF_2α _(1 μM) and endothelial cell network formation (C) and proliferation (D) were assessed. HUVECs were infected with scrambled adenovirus (scr) or short hairpin adenovirus targeted against the FP receptor (sh478) and treated with V CM or P CM. Endothelial network formation (E) and proliferation (F) was determined as described in the methods. (G) HUVECs were incubated with V CM or P CM in the absence/presence of NS398 or indomethacin and network formation was assessed. Data are expressed as fold or percentage increase over control V CM (V CM = 1 or 100%, not shown) and represented as mean ± SEM. (* represents statistical significance P < 0.05).

Since the secretion of PGF_2α _was elevated in HUVECs following P CM treatment, we investigated the role of the F-prostaglandin receptor (FP) in endothelial cell function. We found a significant elevation in endothelial FP receptor expression after 3 hrs of treatment with P CM (Fig. 5A; P < 0.05). To determine if the regulation of FP receptor was mediated by FGF2 in the P CM, we co-incubated HUVECs with P CM in the absence/presence of inhibitors of FGFR1 tyrosine kinase activity (SU4984) or ERK1/2 (PD98059). Incubation of HUVECs with P CM and SU4984 or PD98059 significantly decreased the levels of FP receptor (Fig. 5B; P < 0.05) indicating that FP receptor expression was regulated by FGF2-FGFR1 interaction via the ERK1/2 pathway.

Next, as PGF_2α _secretion and FP receptor expression was increased after P CM treatment, we explored the direct effect of PGF_2α_, acting via the FP receptor, on endothelial cell network formation and proliferation. We found that addition of 1 μM exogenous PGF_2α _significantly increased endothelial cell network formation (Fig. 5C; P < 0.05). In contrast, incubation of HUVECs with 1 μM PGF_2α _had no effect on endothelial cell proliferation (Fig. 5D). To investigate the autocrine/paracrine effect of PGF_2α_-FP receptor interaction in P CM-induced endothelial network formation, we used a short hairpin RNA (shRNA) adenoviral construct targeted against the FP receptor (sh478) to knockdown FP receptor expression in HUVECs. HUVECs were infected with scrambled adenovirus (scr) or sh478 for 24 hrs. Efficiency of the FP shRNA in ablating receptor expression was confirmed in the laboratory by quantitative RT-PCR and Western blot analysis (data not shown). Infection of HUVECs with sh478, significantly reduced P CM-induced network formation compared to scrambled control virus (Fig. 5E, P < 0.05). In contrast, infection of HUVECs with FP receptor shRNA adenovirus did not alter P CM-induced endothelial proliferation compared to HUVECs infected with the control scrambled adenovirus (Fig. 5F). Similar data were obtained using a second shRNA targeted to a different region of the FP receptor (sh306; data not shown). To confirm the role of COX-2 and FP receptor in endothelial cell network formation, HUVECs were treated with P CM in the absence or presence of the COX-2 inhibitor (NS398) or FP receptor antagonist (AL8810) (Fig 5G). Addition of NS398 or AL8810 significantly inhibited P CM-induced endothelial cell network formation (Fig 5G, P < 0.05).

## Discussion

FGF2 is one of 23 fibroblast growth factor family members and signals via one of four receptors, FGFR1, 2, 3 and 4, of which FGFR1 is most commonly expressed on endothelial cells [18]. A principal role of secreted FGF2 is to stimulate blood vessel growth although we have shown previously that it can also act as a potent autocrine growth factor to enhance epithelial cell proliferation [12]. In vitro and animal xenograft studies have shown that secretion of epithelial FGF2 in endometrial adenocarcinoma xenografts can enhance tumour growth by enhancing blood vessel size and width [19]. Furthermore antisense targeting of FGF2 in such model systems is known to reduce tissue microvascular density as well as xenograft size [20].

Endothelial cell differentiation and proliferation are two of the processes required for angiogenesis [11,21-23]. In the present study we have shown that conditioned medium from endometrial adenocarcinoma cells, which stably express the FP receptor to the levels observed in endometrial adenocarcinomas (FPS cells) and produce FGF2, promotes endothelial network formation (differentiation) and proliferation. Using a specific FGFR1 tyrosine kinase inhibitor and FGF2-immunoneutralised conditioned medium, we showed that the effects of conditioned medium on endothelial cell network formation and proliferation were via FGF2-FGFR1 signalling. We found that although the FGF2-immunoneutralised treatment inhibited network formation and proliferation, it was less effective than the FGFR1 inhibitor SU4984. We believe this difference lies in the residual FGF2 remaining in the FGF2-immunoneutralised CM after neutralisation [12] which could, albeit to a lesser extent, activate the FGF receptor on HUVECs.

To explore the signalling pathways activated in HUVECs by FGF2, following its release from epithelial cells in response to PGF_2α_-FP receptor activation, we used small molecule chemical inhibitors of intracellular signalling pathways. We found that conditioned medium from PGF_2α_-treated Ishikawa FPS cells enhanced endothelial cell network formation via FGFR1 and ERK1/2 independently of PI3K and mTOR. This is in agreement with the observations of Kanda et al. [24], who demonstrated in murine brain endothelial cells that FGF2 induced endothelial network formation is not dependent on activation of the mTOR pathway [24] and Sulpice et al [25] who showed that, in adrenal cortex capillary endothelial cells, ERK1/2 phosphorylation induced by recombinant FGF2 is not mediated via the PI3K pathway [25]. Similarly, Peng et al. showed that FGF2 treatment can induce mTOR phosphorylation in HUVECs [26].

In contrast, we found that conditioned medium-induced endothelial cell proliferation was dependent on ERK1/2 signalling to mTOR as endothelial cell proliferation could be inhibited with the ERK1/2 kinase inhibitor PD98059 and rapamycin, but not the PI3K inhibitors wortmannin or LY294002. This is in agreement with previous studies showing that the ERK1/2 inhibitor PD98059 can inhibit FGF2- induced angiogenesis [27] and HUVEC proliferation [28]. Our data indicate that endothelial network formation and proliferation are regulated by distinct signal transduction pathways which are integrated by ERK1/2 signalling.

ERK1/2 is known to be a potent regulator of cell growth, differentiation and development [29]. Once phosphorylated, ERK1/2 can translocate to the nucleus and promote gene transcription [29]. The phosphorylation and activation of ERK1/2 can be modulated via a multitude of intracellular signal transduction pathways. Hence, we investigated conditioned medium signalling to ERK1/2 in HUVECs and found within our experimental paradigm that ERK1/2 was phosphorylated in a time dependent manner. The fact that ERK1/2 is activated by P CM and that the ERK1/2 inhibitor abolished ERK1/2 phosphorylation, as well as endothelial network formation and proliferation, suggests that ERK1/2 could act as a major transcriptional regulator in this model system. This phosphorylation and activation of ERK1/2 was found to be regulated via FGFR1 signalling to c-Src, since co-treatment of HUVECs with P CM and the FGFR1 inhibitor SU4984 or cSrc inhibitor PP2 significantly inhibited ERK phosphorylation. Furthermore ERK1/2 phosphorylation was found to be independent of PI3K and mTOR as neither the PI3K inhibitor LY294002 nor the mTOR inhibitor rapamycin (data not shown) inhibited P CM-induced ERK1/2 phosphorylation. c-Src is a protein tyrosine kinase which co-ordinates a diverse spectrum of receptor-induced signalling to ERK1/2 via the phosphorylation of signalling intermediates such as Ras and Raf [30]. c-Src has been shown to be involved in FGF-2 induced angiogenesis [31] and a recent study has shown that c-Src, Raf and ERK1/2 are essential for HUVEC lumen formation in vitro [32]. These data suggest that the FGF2-FGFR1-c-Src pathway plays a role in the activation of ERK1/2 by P CM treatment.

Following ERK1/2 activation, mTOR has been shown to be regulated via the tuberous sclerosis complex 1 and 2 (TSC1/2 also called Hamartin and Tuberin) [33,34]. Phosphorylation of TSC2 by ERK1/2 results in its dissociation from TSC1 and its subsequent degradation via the ubiquitin pathway. This inactivates the inhibitory effect of TSC1/2 on the mTOR pathway and allows cellular proliferation to proceed [35].

Over the past decade several reports have highlighted the importance of COX enzymes and prostaglandins in regulating vascular function indirectly. This may occur via the activation of ERK1/2 signalling resulting in epithelial or stromal cell production of pro-angiogenic factors which act in a paracrine manner on endothelial cells [4,36,37]. This is in agreement with our observations here whereby FGF2, released by Ishikawa FPS cells in response to PGF_2α_, enhanced the expression of COX-2 in endothelial cells via the FGF2-FGFR1-ERK1/2 pathway. Similarly, FGF2 has been shown to upregulate endothelial COX-2 in murine cerebral microvascular cells leading to an increase in prostaglandin E_2 _production [38].

Prostaglandins have been shown to be secreted by endothelial cells and to influence directly endothelial cell function via their receptors on endothelial cells [39-41]. These studies showed that PGE_2 _present in the endothelial environment can enhance endothelial cell functions [38-41], however in our study we found no significant elevation in PGE_2 _biosynthesis in response to P CM. Instead, we found that endothelial cells secrete elevated levels of PGF_2α _following activation by CM from PGF_2α_-treated Ishikawa FPS cells and that this PGF_2α _secretion was regulated via the FGF2-FGFR1-ERK1/2-mediated induction of COX-2 since the specific COX-2 inhibitor significantly reduced PGF_2α _secretion. In order to determine whether COX-1 contributed towards the generation of PGF_2α_, a general COX inhibitor indomethacin was used. Co-treatment of cells with indomethacin significantly reduced PGF_2α _secretion to a level below that observed for the specific COX-2 inhibitor suggesting that basal levels of COX-1 may, to a lesser extent, contribute towards the secretion of PGF_2α_. These data indicate that although PGE_2 _is secreted in higher quantities than PGF_2α _by unstimulated HUVECs [42], under P CM stimulated conditions, prostaglandin F_2α _is the predominant COX-2 product. Furthermore, this suggests that the endothelial signalling pathways induced by FGF2 are context dependent, i.e. dependent on the nature of the external stimulus, such as cancer conditioned medium, from which the FGF2 originates. We found exogenous prostaglandin F_2α _was able to stimulate endothelial cell network formation but not proliferation. Interestingly, the effect of exogenous PGF_2α _on network formation was less than that observed for P CM. We believe that the higher levels of FP receptor in endothelial cells induced by P CM accounts for this difference. It is likely that the upregulated FP receptor in P CM treated HUVECs would enable a greater signalling capacity and ability to form networks compared with HUVECs treated with exogenous PGF_2α _alone in the absence of growth factors, where FP receptor expression is not induced. Using a specific FP receptor short hairpin RNA (FP shRNA) in an adenoviral delivery system for targeted ablation of endothelial FP receptor, we found that P CM-induced endothelial network formation was regulated by the endothelial FP receptor. In addition, the use of a chemical inhibitor against COX-2 and a specific FP receptor antagonist further confirmed a role for endothelial PGF_2α _signalling through the endothelial FP receptor in the regulation of P CM-induced endothelial cell network formation.

## Conclusions

**Figure 6:**
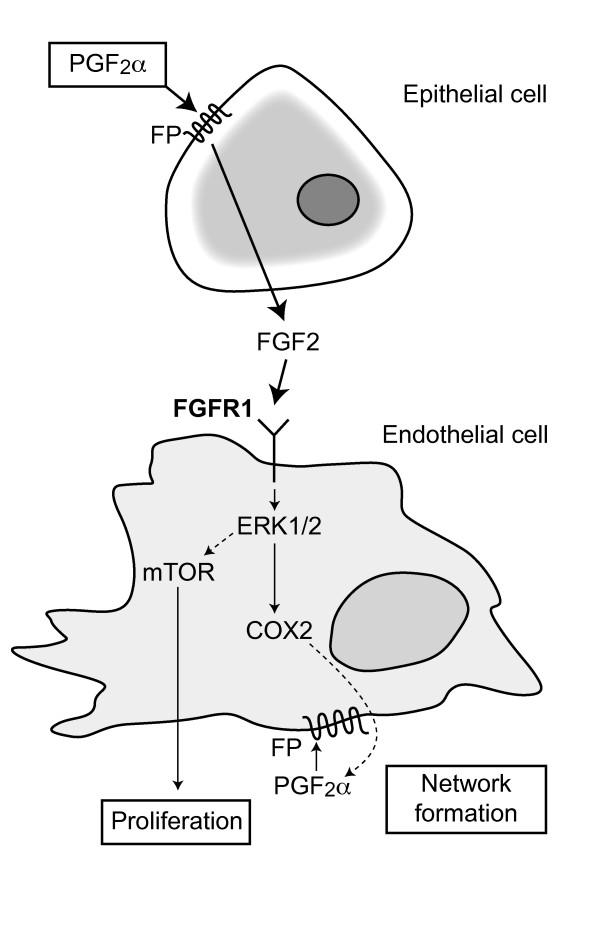
**A schematic representation of PGF_2α_-FP receptor signalling which differentially regulates endothelial network formation and proliferation**. PGF_2α_-FP receptor signalling in epithelial cells promotes the release of FGF2 into culture medium (conditioned medium). Conditioned medium treatment of HUVECs promotes the activation of ERK1/2 signalling via the FGF2-FGFR1. ERK1/2 activates divergent signalling pathways to endothelial proliferation and network formation. Endothelial proliferation is activated via mTOR. Endothelial network formation is activated via the induction of endothelial COX-2 enhancing the biosynthesis and release of PGF_2α_. Endothelial PGF_2α _subsequently acts on endothelial FP receptors to promote network formation (endothelial differentiation).

As summarised in fig. 6, our data show that PGF_2α_-FP receptor signalling in endometrial adenocarcinoma cells produces FGF2, which acts in a paracrine manner on endothelial FGFR1 to promote endothelial cell differentiation and proliferation via distinct intracellular mechanisms. We demonstrate a novel mechanism whereby FGF2 induces the secretion of prostaglandin F_2α _to regulate P CM-induced endothelial cell differentiation. This the first study to show, with FP shRNA, that endothelial FP receptors can mediate endothelial cell differentiation but not proliferation. We believe that these findings have relevance for endometrial pathologies, such as endometrial adenocarcinoma, which have aberrant expression of FP receptor and we propose a molecular mechanism whereby FP receptor may regulate vascular function [13,43]. Furthermore our data suggest that targeted antagonism of epithelial FP receptor signalling, to reduce the production of growth factors, or endothelial FP receptor signalling, to prevent differentiation of endothelial cells, could provide an anti-angiogenic approach to reducing tumour vasculature and growth.

## Methods

### Reagents

The FGF2 antibody recognising the 18 kDa isoform of FGF2 (sc1360) was purchased from Santa Cruz Biotechnology (Autogen-Bioclear, Wiltshire, UK). Arachidonic acid (AA), PGF_2α _and indomethacin were purchased from Sigma Chemical Co. (Dorset, UK). PD98059, SU4984, LY294002, PP2, NS398, wortmannin and rapamycin were purchased from Calbiochem (Nottingham, UK). Recombinant FGF2 peptide was purchased from PeproTechEC Ltd. (London, UK). Anti-phospho-p42/44 ERK and anti-p42/p44 ERK were purchased from Cell Signalling Technologies/New England Biolabs (Hertfordshire, UK).

### Cell culture

Ishikawa cells stably expressing FP receptor (Ishikawa FPS cells) were cultured in Dulbecco's Modified Eagle's Medium (DMEM, Invitrogen, Paisley, UK) with 10% foetal bovine serum (FBS) and 1% penicillin/streptomycin as described previously [13]. Human umbilical vein endothelial cells (HUVECs) (Lonza, Walkersville, USA) were cultured in Endothelial Basal Medium (EBM-2) with 2% FBS and growth supplements (VEGF, FGF, PGDF, IGF, EGF, ascorbic acid, heparin and gentamycin) subsequently referred to as Endothelial Growth Medium (EGM-2) (Lonza, Walkersville, USA). Under experimental conditions HUVECs were incubated with EBM-2 containing 1% fetal bovine serum (FBS) with the addition of ascorbic acid and gentamycin (EBM1%) (Lonza, USA). Cell viability in the presence of chemical compounds used to inhibit specific signal transduction pathways was assessed using the CellTitre96AQueous One Solution Proliferation Reagent (Promega, Southampton, UK).

### Conditioned medium

Conditioned medium (CM) was prepared as described previously [12]. Briefly, FPS cells were seeded at a density of 2 × 10^6 ^cells and allowed to adhere before serum-starvation for 24 hrs. Thereafter, cells were treated with 20 mls of DMEM containing 8.4 μM indomethacin in the presence of 100 nM PGF_2α _or vehicle for 24 hrs to create PGF_2α _conditioned medium (P CM) or vehicle conditioned medium (V CM). Conditioned medium from three independent experiments was pooled, aliquoted and stored at -20°C until required. FGF2 was immunoneutralised from the PGF_2α _conditioned medium by overnight incubation with 0.5 μg/ml FGF2 antibody. The immune complex was removed by 4 hr incubation with 20 μl of a 50% protein A plus G slurry (Calbiochem). Conditioned medium immunoneutralised with Goat IgG was used as a control. Immunoneutralised CM was aliquoted and stored at -20°C until use. The FGF2 content in the CM before and after immunoneutralisation was confirmed by ELISA as described previously [12].

### Network assays

Network assays were carried out using 12-well Transwell plates (Corning Costar, Cambridge, UK). The upper chambers were coated with 80 μl of growth factor (GF)-reduced Matrigel (BD Biosciences, MA, USA) in the absence/presence of SU4984 (20 μM), PD98059 (50 μM), rapamycin (100 ng/ml), wortmannin (200 nM), LY294002 (50 μM), NS398 (10 μM) or AL8810 (50 μM) and incubated at 37°C for 30 mins to allow thin gel formation. HUVECs were plated onto the gel (2.5 × 10^4 ^cells/well) in EBM 1%. In the lower chamber V CM or P CM was added. Transwell plates were incubated at 37°C in a 5% CO_2 _atmosphere for 16 hrs. Subsequently, cell networks were fixed with 100% ice cold methanol and stained with haematoxylin. To assess network formation, each well was divided into 5 sections. Hotspots of each section were photographed, 5 photos per well at ×10 magnification, using an inverted microscope and camera (Axiovert 200, Carl Zeiss, Germany). The number of network branches was counted blind. Experiments were repeated at least four times in duplicate. Fold difference was determined by dividing the value obtained from P CM treated cells by the value obtained from V CM treated cells. Data were transformed to percentage increase in network formation with V CM = 100% and are presented as mean ± SEM.

### Proliferation assay

HUVECs were seeded in 96-well plates at 3000 cells/well. Following attachment, cell medium was replaced with EBM1% for 3 hours. Cells were then treated with CM, diluted 1:1 (v/v) with EBM1%, in the absence/presence of SU4984, PD98059, rapamycin, wortmannin, LY294002 or FGF2-immunoneutralised CM. Treatments were replaced three times during the 96 hr incubation. Proliferation was determined using the CellTitre96AQueous One Solution Proliferation Reagent (Promega) as per the manufacturer's instructions. The experiments were repeated three times in quadruplicate. Fold difference was determined by dividing the absorbance obtained by P CM treated cells by the absorbance obtained by V CM treated cells. Data were transformed to percentage increase in proliferation with V CM = 100% and are presented as mean ± SEM.

### Western Blot analysis

HUVECs were seeded at 2 × 10^5 ^cells per 60 mm diameter dish and left to adhere for 24 hrs before GF-starvation overnight. The next day, HUVECs were treated with P CM or V CM for the time indicated in the figure legend, rinsed with ice-cold phosphate-buffered saline and lysed for 20 mins with protein lysis buffer containing inhibitor cocktail mix as described previously [43]. Protein concentration was determined with a protein assay (BioRad, Hercules, CA) and approximately 16 μg of protein was resolved and immunoblotted as previously described [44]. Immunoblots were blocked in Odyssey Blocking buffer™ (LI-COR Biosciences, Cambridge, UK) before overnight incubation with primary phospho-p42/44 and p42/44 antibodies (diluted 1:1000 in Odyssey blocking buffer) at 4°C. The following day, blots were washed and incubated with the goat anti-mouse IRDye™ 800 (1:10,000) (Rockland Immunochemicals Inc., Gilbertsville, PA, USA) and goat anti-rabbit Alexafluor 680 (1:5000) (Invitrogen) for 60 minutes at room temperature. Immunoreactive proteins were detected and quantified using the Odyssey infrared imaging system (LI-COR Biosciences). ERK1/2 phosphorylation was calculated by dividing the value obtained from the phosphorylated ERK1/2 channel (700 nm) by the value obtained from total ERK1/2 channel (800 nm) and expressed as fold above vehicle controls. Results are expressed as mean ± SEM from at least four independent experiments.

### Taqman quantitative RT-PCR

Taqman RT-PCR was performed as described previously using sequence specific primers and probes designed to span an intron [43,45,46]. Briefly, HUVECs were seeded at 5 × 10^4 ^cells per well with EBM1% in a 6 well plate. Following overnight serum starvation, cells were treated with V CM or P CM, diluted 1:1 (v/v) with EBM 1% in the absence or presence of SU4984 (20 μM) or PD98059 (50 μM) for the time indicated in the figure legends. RNA was extracted, reverse transcribed and RT-PCR performed using the ABI Prism 7900 as described previously [12]. COX-1, COX-2 and FP mRNA were normalized using ribosomal 18S as an internal control. Experiments are representative of at least five independent experiments. Fold difference was determined by dividing the value obtained from P CM treated cells by the value obtained from V CM treated cells and represented as mean ± SEM.

### PGF_2α_/PGE_2 _ELISA

HUVECs were seeded at a density of 5 × 10^4 ^cells per 35 mm diameter dish and serum starved overnight. Thereafter cells were treated with V CM or P CM diluted 1:1 (v/v) with EBM 1% containing 3 μg/ml arachidonic acid for the time indicated in the figure legend. After treatment CM was collected and stored at -20°C until required. PGF_2α _and PGE_2 _secretions in the culture media were assayed by ELISA as described previously [47,48]. When using chemical inhibitors, cells were preincubated with EBM1% and NS398 (10 μM) or indomethacin (8.4 μM) for 30 minutes and subsequently, cells were treated with V CM or P CM in the absence or presence of inhibitors with the addition of 3 μg/ml arachidonic acid for 6 hrs. Experiments were repeated three times. The data are presented as mean ± SEM.

### Short hairpin DNA constructs and preparation of adenoviral stocks

To generate FP receptor knockdown vectors, oligonucleotides encoding short hairpin transcripts were annealed and individually cloned into the adenovirus shuttle vector pDC316. The start codon of the FP receptor (NM_000959) was used as a reference and labelled as basepair 1. Thus the target sequences corresponded to 478 bp downstream and included a scrambled (scr) negative control: Sh478: 5'-GTGGCCTGGTAATCACTGA; scr: 5'-TTACTCGACGCATGTGCTT. High titre stocks (<10e10 viral plaque forming units/ml, pfu/ml) were prepared using the AdMax system (Microbix Biosystems Inc., Toronto, Ontario, Canada). Briefly, an adenovirus genomic plasmid (pBHGLoxdeltaE1,3Cre) was cotransfected with the shuttle vector into HEK293 cells. After 10 days, plaques were purified and seeded into a T75 flask of HEK293 to generate the first seed. When HEK293 cells showed a cytopathic response; virus was released from the cells by three cycles of freeze thawing. This starter culture was used to produce large bulk preparations of adenovirus. Viral particles were purified using a Vivascience AdenoPack column (Generon House, Eton Wick, UK), buffer exchanged into 8 volumes of 2.5% glycerol, 20 mM Tris-HCl (pH 8) and then concentrated. The viral titre was determined using a modified Adeno-X rapid titre kit (Clontech-Takara Bio Europe, Saint-Germain-en-Laye, France) with 1:1000 rabbit anti-adenovirus Serotype 5 hexon antisera (Labfrontier, Seoul, Korea).

To ablate FP receptor expression, HUVECs were seeded at a density of 3 × 10^5 ^cells/25 cm^2 ^in EGM-2 and incubated with 100 viruses (either scr or sh478) per cell (MOI) for 24 hrs at 37°C in a 5% CO_2 _atmosphere. The next day, the viral infected cells were washed, trypsinised, counted and used in the network formation and proliferation assays.

### Statistics

Statistical significance was assessed on untransformed data with one-way ANOVA and Dunnett's post hoc test using Prism 5.0 (Graph Pad, San Diego, CA). A *p*-value of less than 0.05 (P < 0.05) was considered to be statistically significant.

## Authors' contributions

MCK carried out the main part of the experiments, participated in the design, statistical analysis, drafting and writing of the manuscript. PB produced the short hairpin DNA constructs and preparation of adenoviral stocks. HNJ conceived the study and helped to draft the manuscript. KJS conceived the study, and participated in its design and coordination and helped to draft the manuscript. All authors read and approved the final manuscript.
